# The Biosynthesis of Phenolic Compounds Is an Integrated Defence Mechanism to Prevent Ozone Injury in *Salvia officinalis*

**DOI:** 10.3390/antiox9121274

**Published:** 2020-12-14

**Authors:** Alessandra Marchica, Lorenzo Cotrozzi, Rebecca Detti, Giacomo Lorenzini, Elisa Pellegrini, Maike Petersen, Cristina Nali

**Affiliations:** 1 Department of Agriculture, Food and Environment, University of Pisa, Via del Borghetto 80, I-56124 Pisa, Italy; alessandra.marchica@agr.unipi.it (A.M.); lorenzo.cotrozzi@agr.unipi.it (L.C.); rebeccadetti@gmail.com (R.D.); giacomo.lorenzini@unipi.it (G.L.); cristina.nali@unipi.it (C.N.); 2 CIRSEC, Centre for Climate Change Impact, University of Pisa, Via del Borghetto 80, I-56124 Pisa, Italy; 3 Nutrafood Research Center, University of Pisa, Via del Borghetto 80, I-56124 Pisa, Italy; 4 Institut für Pharmazeutische Biologie und Biotechnologie, Philipps-Universität Marburg, Robert Koch Str. 4, D-35037 Frankfurt, Germany; petersen@staff.uni-marburg.de

**Keywords:** antioxidants, caffeic acid, flavonoids, hydroxybenzoic acids, hydroxycinnamic acids, PAL, phenylpropanoids, rosmarinic acid, sage, secondary metabolites

## Abstract

Specialized metabolites constitute a major antioxidant system involved in plant defence against environmental constraints, such as tropospheric ozone (O_3_). The objective of this experiment was to give a thorough description of the effects of an O_3_ pulse (120 ppb, 5 h) on the phenylpropanoid metabolism of sage, at both biochemical and molecular levels. Variable O_3_-induced changes were observed over time among the detected phenylpropanoid compounds (mostly identified as phenolic acids and flavonoids), likely because of their extraordinary functional diversity. Furthermore, decreases in the phenylalanine ammonia-lyase (PAL), phenol oxidase (PPO), and rosmarinic acid synthase (RAS) activities were reported during the first hours of treatment, probably due to an O_3_-induced oxidative damage to proteins. Both PAL and PPO activities were also suppressed at 24 h from the beginning of exposure, whereas enhanced RAS activity occurred at the end of treatment and at the recovery time, suggesting that specific branches of the phenolic pathways were activated. The increased RAS activity was accompanied by the up-regulation of the transcript levels of genes like *RAS*, tyrosine aminotransferase, and cinnamic acid 4-hydroxylase. In conclusion, sage faced the O_3_ pulse by regulating the activation of the phenolic biosynthetic route as an integrated defence mechanism.

## 1. Introduction

Plants, being sessile organisms, are persistently exposed to environmental stresses, which usually have negative impacts on their growth and productivity [1]. Therefore, plants have developed various adaptation mechanisms that can be activated to sustain their life cycle under environmental challenges [2]. Plants possess a forceful and multifarious antioxidant system composed of enzymatic reactions (e.g., superoxide dismutase, catalase, ascorbate peroxidase) and non-enzymatic compounds (e.g., ascorbic acid and glutathione), which are involved in detoxification, removal, and/or neutralization of reactive oxygen species (ROS) overproduction due to biotic and abiotic stresses [3].

Secondary metabolites—classified into, e.g., terpenoids, phenylpropanoids, and nitrogen containing compounds, based on their biosynthetic origin [4]—are well suited to constitute a major antioxidant system with a central role in plant defence against environmental constraints by (i) avoiding the generation of ROS (e.g., catalyzing oxygenation reactions through formation of metallic complexes reducing/inhibiting the activities of oxidizing enzymes), and (ii) quenching ROS once they are formed [5]. These specialized molecules are produced by plants to respond to a large number of diverse signals, both internal ones and those emanating from the environment, which are critical for their survival and adaption as sessile organisms. Accumulation of these compounds is usually a consistent feature in the plant defence mechanisms as they can increase the tolerance and adaptability to different stresses [5].

**Figure 1:**
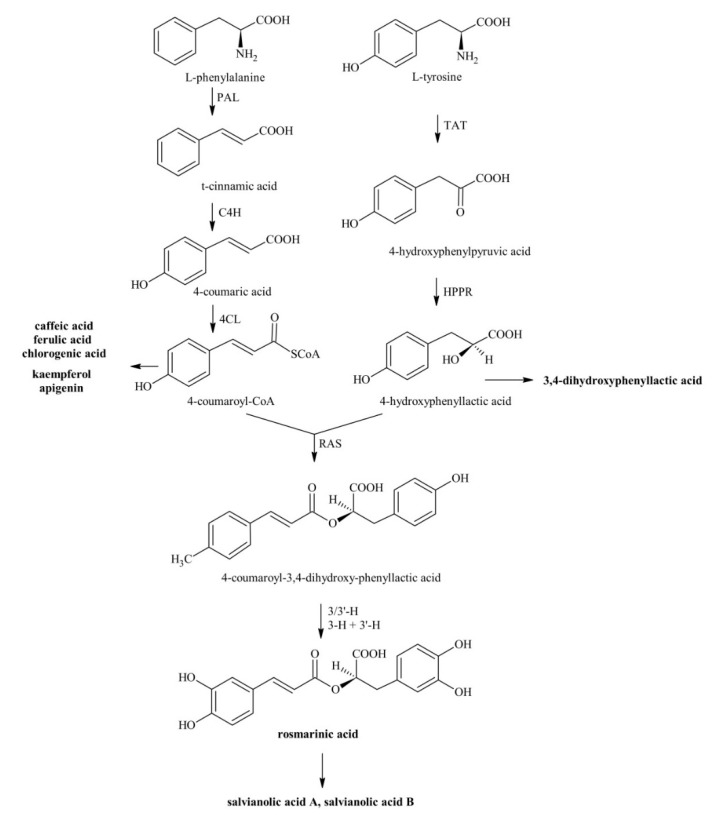
Intermediates and enzymes of the biosynthesis of rosmarinic acid and related phenolic compounds; PAL = phenylalanine ammonia-lyase, C4H = cinnamic acid 4-hydroxylase, 4CL = 4-coumarate CoA-ligase, TAT = tyrosine aminotransferase, HPPR = hydroxyphenylpyruvate reductase, RAS = rosmarinic acid synthase, 3-H, 3′-H, 3/3′-H = 3-/3′-hydroxylases.

Phenolics are the largest group of plant secondary metabolites. Varying from simpler aromatic compounds to more complex ones, they mainly derive from the shikimate/phenylpropanoid pathway [6]. They are classified as hydroxybenzoic and hydroxycinnamic acid derivatives based on their distinctive carbon frameworks as well as on the position and number of hydroxyl groups on the aromatic ring [7]. Hydroxycinnamic acids, which are derivatives of cinnamic acid having a C6-C3 framework, are more prevalent in nature than hydroxybenzoic acids, which instead are derivatives of benzoic acid with a C6-C1 structure. Hydroxycinnamic acids are also more efficacious antioxidants than hydroxybenzoic acids because of the presence in their structure of the –CH=CH–COOH group, instead of the –COOH group [8]. The accumulation of phenolics in plant tissues can be reached in two ways: (i) reducing their consumption because the oxidative pressure is low, and/or (ii) enhancing their biosynthetic route in an integrated defence mechanism through increased performance of enzymes, such as phenylalanine ammonia-lyase (PAL) and chalcone synthase (CHS), accompanied by the up-regulation of the transcript levels of genes encoding key biosynthetic enzymes like PAL, cinnamic acid 4-hydroxylase (C4H), 4-coumarate CoA-ligase (4CL), tyrosine aminotransferase (TAT), and rosmarinic acid synthase (RAS) (Figure 1 [4,9]).

*Salvia* (sage), the largest genus of the Lamiaceae family, broadly cultivated in the Mediterranean basin, is a rich source of phenylpropanoids [10]. Caffeic acid derivatives play a major role among the specialized metabolites of sage species [11]. Moreover, flavonoids are widely distributed in this genus, mostly present as flavones (e.g., apigenin, luteolin, and their corresponding 6-hydroxylated derivatives), flavonols, and their glycosides (e.g., kaempferol and quercetin) [11]. Flavonoids are known to play a major role in reducing photo-oxidative damage [12] and scavenging ROS [13].

In the context of climate change, the phenylpropanoid pathway is one of the most affected targets of detrimental environment conditions, such as increasing amounts of tropospheric ozone (O_3_), which affect not only the synthesis, but also the accumulation of these metabolites and the induction of related enzymes and genes. Tropospheric O_3_ is one of the most important greenhouse gases involved in global warming, harmful to human health and crop production because of its high oxidative potential [14]. Despite numerous legislatory attempts aimed to control emissions of its precursors, O_3_ is still among the major air pollutants worldwide, especially in areas with elevated temperatures such as the Mediterranean basin, where it frequently exceeds the World Health Organization (WHO) guideline average values of 50 ppb [15]. For example, it reached hourly peaks of 90 ppb during the summer 2019 in Italy [16]. In a previous study [17], our research group demonstrated that *S. officinalis* plants under chronic O_3_ exposure (120 ppb, 5 h day^−1^, for 90 days) were able to activate already after one month of exposure a photoprotection mechanism including carotenoids and phenylpropanoid compounds (even though impairments to plant processes occurred). Although it is known that plant responses to chronic and acute O_3_ may differ [18], to the best of our knowledge, the effects of a single O_3_ pulse on *S. officinalis* have not been investigated, except by Marchica et al. [19], using a very high O_3_ concentration (i.e., 200 ppb).

For these reasons, the primary objective of the present experiment was to give a thorough description of the effects of an O_3_ pulse at a lower concentration (120 ppb) on the phenylpropanoid metabolism of *S. officinalis* at both biochemical and molecular levels, also focusing on the expression of genes and on enzyme activities related to the biosynthesis of rosmarinic acid. Specifically, we asked the following questions: (i) What is the potential role of phenolics (e.g., caffeic acid derivatives) and flavonoids in regulating the responses to a single O_3_ pulse? (ii) Which enzymes involved in the phenolic pathway are more affected by high O_3_ levels, and why? (iii) Which molecular alterations of gene expression related to the biosynthesis of rosmarinic acid are induced by O_3_ treatment?

## 2. Materials and Methods

### 2.1. Experimental Design

Experimental activities were performed at the field-station of San Piero a Grado (Pisa, Italy; 43°40′48″ N, 10°20′48″ E, 2 m a.s.l.) owned by the Department of Agriculture, Food, and Environment of the University of Pisa. In May 2019, 30 seedlings of *S. officinalis* (8 months old, 1–3 stems per seedling), grown under field conditions in plastic pots (3.7 L volume) containing a mixture of peat and steam-sterilized soil (1:1, *v*/*v*), were selected for uniformity of size (approximately 30 cm tall), distributed among four chambers placed inside a greenhouse (day and night mean temperatures were 28 and 20 °C, respectively, and maximum day and night relative humidity were approximately 60 and 50%, respectively) under charcoal-filtered air. After ten days, half of the plants were exposed to a target O_3_ concentration of 120 ppb (1 ppb = 1.96 µg m^−3^, at 25 °C and 101.325 kPa) for 5 h from 10:00 to 15:00, while the other half of the plants were maintained under charcoal-filtered air containing negligible O_3_ concentrations (controls). Ozone was generated by a Fisher 500 air-cooled apparatus (Fisher America Inc., Houston, TX, USA), supplied with pure oxygen, and mixed with inlet air entering the fumigation chamber; its concentration was continuously measured inside the chambers at plant height with a Serinus 10 analyzer (Ecotech Acoem Group, Milan, Italy), as described by Marchica et al. [19]. At 0, 1, 2, 5, and 24 h from the beginning of exposure (FBE), the three youngest fully expanded leaves per plant were harvested on three plants per treatment per sampling time, flash frozen in liquid nitrogen, ground, and stored at −80 °C until biochemical and molecular analyses.

### 2.2. Biochemical Analyses

#### 2.2.1. Metabolites Involved in the Phenylpropanoid Pathway

Phenylpropanoids were analyzed by ultra-high pressure liquid chromatography (UHPLC) using a UHPLC Dionex UltiMate 3000 system equipped with an Acclaim 120 C18 column (5 μm particle size, 4.6 mm internal diameter × 150 mm length) mounted in a Dionex TCC-100 column oven, and a Dionex UVD 170U UV-Vis detector (Thermo Scientific, Waltham, MA, USA). Around 45 mg (fresh weight, FW) of leaf material was extracted in 1 mL of 70% HPLC-grade methanol for three times, with the third extraction performed after an overnight incubation at 4 °C. At each of the three times, extracts were centrifuged for 20 min at 16,000× *g* at 20 °C, and supernatants were collected and combined [20]. Then, the mixed supernatants were washed with hexane and filtered through 0.2 µm Minisart^®^ SRT 15 aseptic filters. Setting the column oven at 30 °C and the flow rate at 1 mL min^−1^, phenylpropanoids were eluted as follows: 100% solvent A (water/methanol/acetic acid, 75:20:5 (*v*/*v*/*v*) for 1 min, a 30 min linear gradient to 100% solvent B (water/methanol/acetic acid, 50:45:5, (*v*/*v*/*v*), a 5 min linear gradient to 100% solvent A, and finally 5 min 100% solvent A. Phenolic compounds were detected at 250, 280, and 350 nm [21]. Compound concentrations were quantified using equations developed linearly relating peak areas with known amounts of pure standards injected into the UHPLC system. Chromatographic data were processed and recorded by Chromeleon Chromatography Management System software, version 6.60-2004 (Thermo Scientific).

#### 2.2.2. Enzymes Involved in Phenolic Metabolism

For protein extraction, leaf material (50 mg FW) was ground to powder in liquid nitrogen and added to 6 mg Polyclar 10 and 1.2 mL 0.5 M Tris-HCl buffer (pH 10.0) containing 5 mM dithiothreitol and mixed thoroughly. After centrifugation (10,000× *g* for 20 min at 4 °C), the protein extract was passed through a PD-10 column (GE Healthcare, Chicago, IL, USA) and kept on ice.

Phenylalanine ammonia-lyase (PAL, EC 4.3.1.24) activity was determined by separation and quantification of the formed reaction product *t*-cinnamic acid by HPLC (Hitachi, Merck, Darmstadt, Germany) and photometric detection at 290 nm according to Dӧring et al. [9]. The PAL assay consisted of 100 μL boric acid/borate buffer (0.078 M H_3_BO_3_, 0.03 M Na_2_B_4_O_7_, and 0.02 M NaCl) pH 8.8, 50 μL 0.1 M l-phenylalanine dissolved in the same buffer, and 100 μL protein extract (see above). After incubation for 30 min at 50 °C, the reaction was stopped by adding 50 μL 6 N HCl. Reaction products were extracted twice by mixing with 500 μL ethyl acetate, centrifugation at 16,000× *g* for 5 min, and collection of the organic phases. After evaporating the solvent, residues were dissolved in 100 μL 50% methanol/0.01% H_3_PO_4_.

Polyphenol oxidase (PPO, EC 1.10.3.2) activity was determined by HPLC (as elaborated below) with photometric detection at 333 nm, according to Leuschner [22]. This assay consisted of 445 μL 0.1 M Na-acetate buffer (pH 4.5), 30 μL 1 M ascorbic acid/sodium ascorbate (pH 4.5), 10 μL 10 mM 4-coumaric acid, and 15 μL protein extract. After incubation for 10 min at 30 °C, the reaction was stopped by adding 50 μL 6 N HCl. Reaction products were extracted as reported above.

Rosmarinic acid synthase (RAS, EC 2.3.1.140) activity was determined by separation and quantification of the hydroxycinnamic acid esters by HPLC (as elaborated below) with photometric detection at 333 nm, according to Berger et al. [23]. This assay consisted of 75 μL 0.1 M potassium phosphate buffer (pH 7.0), 10 μL 12.5 mM ascorbic acid, 10 μL 2.5 mM caffeoyl-CoA, 10 μL 10 mM 4-hydroxyphenyllactate (pHPL, in 20% ethanol), and 20 μL protein extract. After incubation for 2 h at 30 °C, the reaction was stopped by adding 20 μL 6 N HCl. The extraction of the hydroxycinnamic acid esters was performed with ethyl acetate as described above and the dry samples were dissolved in 100 μL 50% methanol/0.01% H_3_PO_4_.

Isocratic HPLC analysis of the extracted reaction products of the PAL, PPO, and RAS enzyme activity assays was performed using an Equisil ODS (250 mm length, 4 mm diameter with a 20 mm pre-column; Dr. Maisch GmbH, Ammerbuch, Germany). The eluents consisted of 50, 40, and 50% methanol/0.01% H_3_PO_4_, respectively, eluted at a flow rate of 1 mL min^−1^. *t*-Cinnamic acid was detected at 290 nm, and caffeic acid and hydroxycinnamic acid esters at 333 nm.

### 2.3. Molecular Analyses

#### 2.3.1. qRT-PCR Primer Design

Specific primers for sage [elongation factor (EF)1α, actin, PAL, C4H, TAT, and RAS] were constructed on homologous sequences selected after BLAST analysis, using as a query *Melissa officinalis* and *Salvia milthiorriza* (GenBank acc. No. FN665700, DQ355979, JN863949, FR670523, KM575933, HM231319, and HM051058). After CLUSTALW multiple-sequence alignment, primers were designed using Primer3 software (230 bp maximum length, optimal melting temperature at 59.3 °C, GC content between 45 and 60%; Applied Biosystems, Foster City, CA, USA; Table 1).

#### 2.3.2. RNA Extraction and Relative Expression Analyses

Total RNA was extracted from frozen (−80 °C) leaf tissue using the RNeasy^®^ Mini Plant Kit (Qiagen Benelux B.V., Venlo, The Netherlands) treated with Amplification Grade DNase I (Sigma-Aldrich, St. Louis, MO, USA) and reverse-transcribed into cDNA (400 ng per sample) using the iScript cDNA synthesis kit (BioRad, Hercules, CA, USA) following the manufacturer’s instructions. Elongation factor 1α was used as a reference gene after confirmation of its transcriptional stability in our experimental conditions and synthesized by Eurofins MWG Operon (Ebersberg, Germany). Each 12 μL qRT-PCR assay contained 5 μL cDNA (diluted 1:20), 0.25 μL of each primer (10 μM), 0.5 μL nuclease-free water, and 7 μL PerfeCTa^®^ SYBR Green SuperMix (Quantabio, Beverly, MA, USA), following the manufacturer’s instructions. qRT-PCR analyses were performed in a PikoReal 96 (Thermo Fisher). The three-step thermal profile comprised three segments: (1) 95 °C/2 min, (2) 40 cycles of 95 °C/15 s, 55 °C/45 s, 68 °C/60 s. Three technical replicates were analyzed for each biological sample. The efficiency of each primer was evaluated by linear regression analysis of serial dilutions of cDNA. The expression level of the four target genes was individually normalized to the transcript level of EF1α and actin, and calculated by the Pfaffl method [24].

### 2.4. Statistical Analyses

Three plants per treatment per time were used (the three leaves sampled from each plant were analyzed separately and then averaged to keep the plant as a statistical unit). The normality of data was preliminarily tested by the Shapiro-Wilk test. The effects of O_3_, time, and their interaction on leaf parameters were analyzed using a full factorial two-way analysis of variance (ANOVA). Comparisons among means were determined by the least significant Tukey’s honestly significant difference (HSD) post-hoc test. Effects with *p*  ≤  0.05 were considered statistically significant. Statistical analyses were performed in JMP 11 (SAS Institute Inc., Cary, NC, USA).

## 3. Results

### 3.1. Metabolites Involved in the Phenylpropanoid Pathway

**Figure 2:**
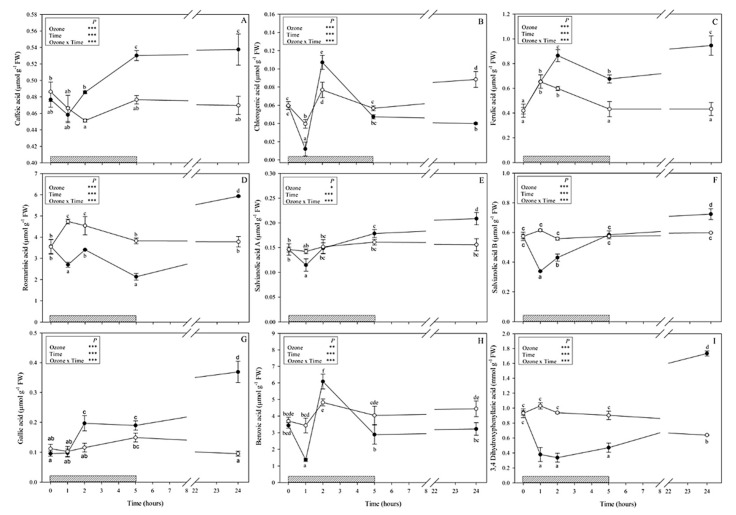
Content of phenolic acids (caffeic acid (**A**), chlorogenic acid (**B**), ferulic acid (**C**), rosmarinic acid (**D**), salvianolic acid A (**E**)and salvianolic acid B (**F**), gallic acid (**G**), benzoic acid (**H**), 3,4 dihydroxyphenyllatic acid (**I**)) (μmol g^−1^ FW) in leaves of *Salvia officinalis* exposed to ozone (120 ppb, 5 h; closed circle) or maintained under filtered air (controls; open circle). Measurements were carried out at 0, 1, 2, 5, and 24 h from the beginning of exposure. Data are shown as mean ± standard deviation (*n* = 3). *p*-values show the results of a full factorial two-way analysis of variance (ANOVA) with O_3_ and time as variability factors (*** *p* ≤ 0.001; ** *p* ≤ 0.01; * *p* ≤ 0.05; ns: *p* > 0.05). For each compound (**A**–**I**), different lowercase letters (a–e) indicate significant differences among means, according to the Tukey’s HSD post-hoc test (*p*  ≤  0.05). FW = fresh weight.

**Figure 3:**
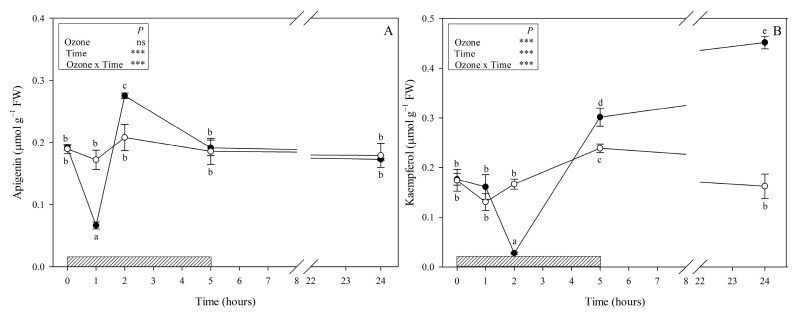
Content of flavonoids (apigenin (**A**), kaempferol (**B**)) (μmol g^−1^ FW) in leaves of *Salvia officinalis* exposed to ozone (120 ppb, 5 h; closed circle) or maintained under filtered air (controls; open circle). Measurements were carried out at 0, 1, 2, 5, and 24 h from the beginning of exposure. Data are shown as mean ± standard deviation (*n* = 3). *p*-values show the results of a full factorial two-way ANOVA with O_3_ and time as variability factors (*** *p* ≤ 0.001; ns: *p* > 0.05). For each compound (**A**,**B**), different lowercase letters (a–e) indicate significant differences among means, according to the Tukey’s HSD post-hoc test (*p*  ≤  0.05). FW = fresh weight.

A total of 11 phenylpropanoids were identified: nine phenolic acids (hydroxycinnamic acids and derivatives: caffeic acid, ferulic acid, chlorogenic acid, rosmarinic acid, salvianolic acids A and B; hydroxybenzoic acids: gallic and benzoic acids; 3,4-dihydroxyphenyllactic acid (danshensu) as tyrosine derived phenolic acid) (Figure 2) and two of the most common flavonoids (apigenin and kaempferol, Figure 3). Rosmarinic and benzoic acids showed higher contents than the other compounds 0.01–1.73 (min-max; all other compounds) vs. 4.2–11.86 and 1.36–6.07 μmol g^−1^ FW (rosmarinic and benzoic acid, respectively). Although no visible symptoms were observed, O_3_ exposure induced significant changes to the phenylpropanoid profile throughout the whole experiment.

Caffeic acid and ferulic acid levels in ozone-treated leaves started to increase at 2 h FBE (+7 and 46%, respectively; Figure 2A,C) and maintained increased levels throughout the whole experiment (+13 and 56% at 5; +15% and 2-fold at 24 h FBE, respectively). Chlorogenic acid showed the lowest concentration of all observed compounds (Figure 2B) and did not show a clear trend; it significantly decreased at 1 h FBE (−67% in comparison with controls), was higher than controls at 2 h FBE (+43%), did not show significant differences between treatments at the end of exposure, and significantly decreased again at the recovery time (−63%). Under O_3_ treatment, the levels of RA, salvianolic acid B, and the tyrosine-derived compound 3,4-dihydroxyphenyllactic acid (danshensu) were strongly reduced at 1 h FBE (−43, −46 and −71% respectively; Figure 2D,F,I), and remained lower than controls until the end of O_3_ treatment (only salvianolic acid B did not show significant differences between treatments at the end of exposure), but they were increased at the recovery time (i.e., 24 h FBE) reaching their maximum values (+57%, +22% and about threefold higher than controls, respectively). Salvianolic acid A in treated plants remained at control levels until the end of exposure and then increased at 24 h FBE (+33% in comparison with controls; Figure 2E). Among hydroxybenzoic acids, gallic acid revealed no significant differences between treated and untreated plants at 1 and 5 h FBE, but it was higher in treated plants than in controls at 2 and 24 h FBE (about two- and fourfold, respectively; Figure 2F). Compared with controls, the levels of benzoic acid of treated plants significantly decreased at 1 h FBE (−60%, Figure 2G), then peaked at 2 h FBE (+26%), and decreased again starting from the end of exposure (−29 and −27% at 5 and 24 h, respectively).

Among the flavonoids, apigenin did not show a clear trend; it significantly decreased at 1 h FBE (−65% in comparison with controls, Figure 3A), increased at 2 h FBE (+35%), and revealed no significant differences between treatments at 5 and 24 h FBE. Kaempferol significantly decreased at 2 h FBE (−88% in comparison with control, Figure 3B) and strongly increased starting from 5 h FBE (+30%), reaching a maximum at the recovery time (about threefold higher than controls).

### 3.2. Enzymes Involved in the Phenylpropanoid Pathway

**Figure 4:**
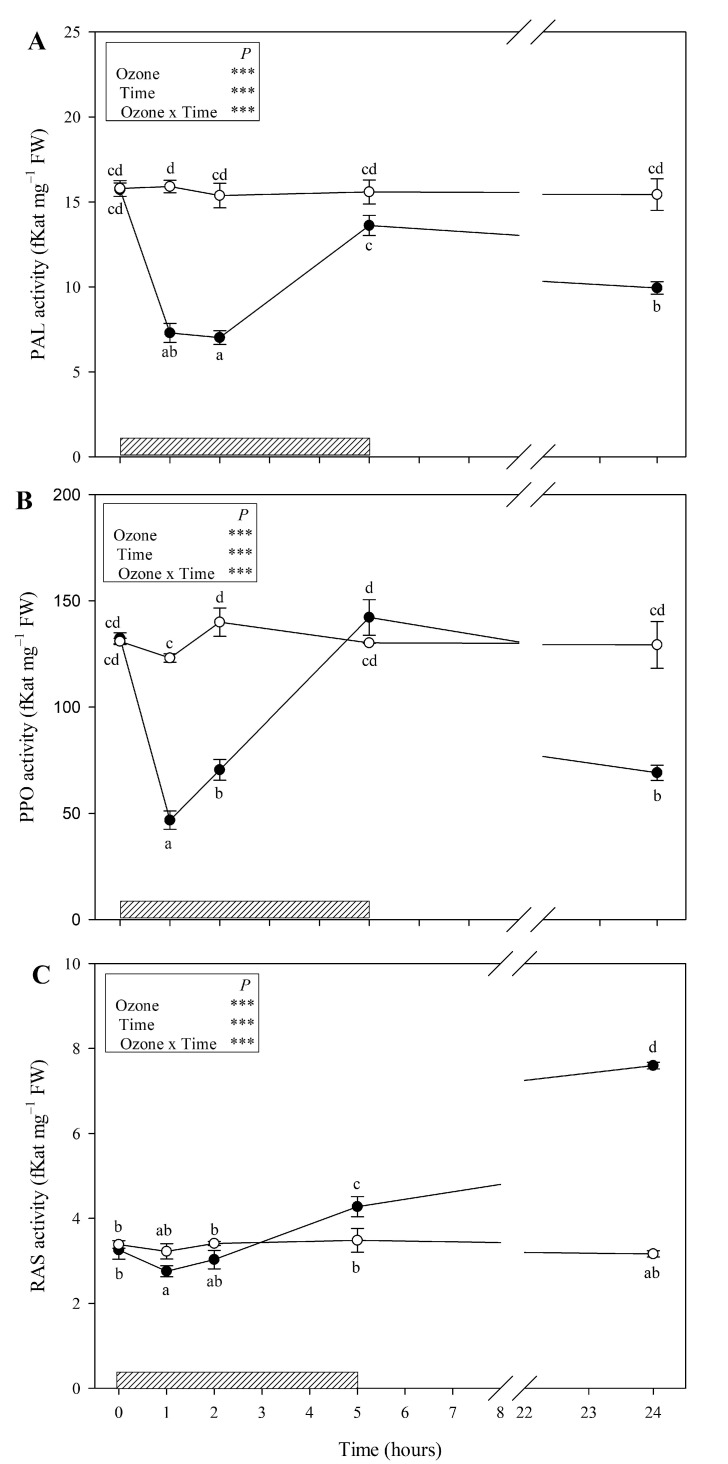
Time course of phenylalanine ammonia-lyase (PAL; (**A**)), polyphenol oxidase (PPO; (**B**)), and rosmarinic acid synthase (RAS; (**C**)) activities in leaves of *Salvia officinalis* exposed to ozone (120 ppb, 5 h; closed circle) or maintained under filtered air (controls; open circle). Data are shown as mean ± standard deviation (*n* = 3). Measurements were carried out at 0, 1, 2, 5, and 24 h from the beginning of exposure. Boxes show the results of a full factorial two-way ANOVA with ozone and time as variability factors (*** *p* ≤ 0.001). For each compound (**A**–**C**), different lowercase letters (a–d) indicate significant differences among means, according to the Tukey’s HSD post-hoc test (*p*  ≤  0.05). The dashed bar indicates the ozone treatment (i.e., 5 h). FW = fresh weight.

Ozone treatment strongly decreased PAL and PPO activities at 1 and 2 h FBE (around −50 and −60% in comparison with controls, respectively; Figure 4A,B). At the end of the O_3_ treatment, the activity of these enzymes came back to control levels and decreased again at 24 h FBE (−36 and −47%, respectively). RAS activity slightly decreased at 1 and 2 h FBE (−15 and −11% in comparison with control, respectively, Figure 4C) and strongly increased again starting from 5 h FBE (+23%), reaching a maximum at the recovery time (+59%).

### 3.3. Gene Expression Analyses

**Figure 5:**
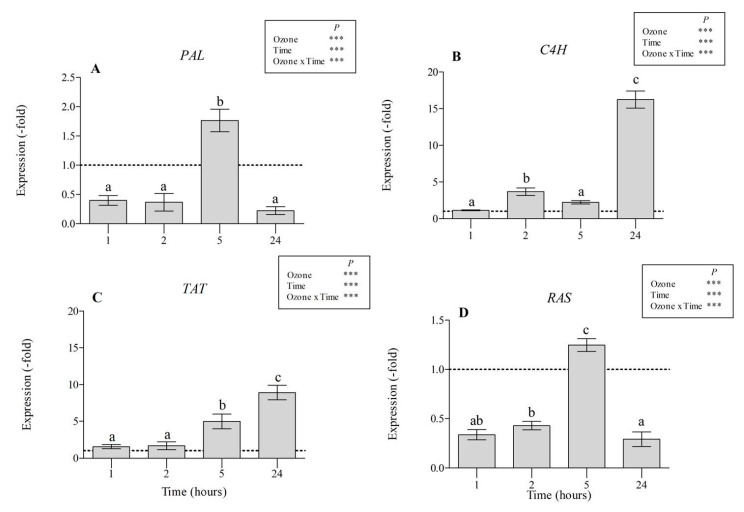
Expression levels of phenylalanine ammonia-lyase (PAL; (**A**)), cinnamic acid 4-hydroxylase (C4H; (**B**)), tyrosine aminotransferase (TAT; (**C**)), and rosmarinic acid synthase (RAS; (**D**)) genes in leaves of *Salvia officinalis* exposed to ozone (120 ppb, 5 h). Gene expression in plants maintained under filtered air (controls) was set to the value 1.0 (dashed line). Data are shown as mean ± standard deviation (*n* = 3). Measurements were carried out at 1, 2, 5, and 24 h from the beginning of exposure. Different letters indicate significant differences (*p* ≤ 0.05). Boxes show the results of a full factorial two-way ANOVA with ozone and time as variability factors (*** *p* ≤ 0.001). For each compound (**A**–**C**), different lowercase letters (a–c) indicate significant differences among means, according to the Tukey’s HSD post-hoc test (*p*  ≤  0.05).

At the molecular level, the effect of O_3_ was investigated by monitoring the transcript abundance of the PAL, C4H, TAT, and RAS genes. In treated plants, a decrease of the transcript levels in comparison with controls was observed at 1 and 2 h FBE for PAL (−40 and −36%, respectively; Figure 5A) and RAS (−34 and 43%, respectively; Figure 5D), indicating that they were downregulated by O_3_ or, alternatively, the mRNAs could have been degraded. By contrast, PAL and RAS gene expression was upregulated at the end of treatment (about 2-fold higher than controls), but it decreased again at 24 h FBE (−22 and −29%, respectively). Conversely, C4H and TAT mRNA levels started to increase at 2 h FBE (about twofold higher than controls; Figure 5B,C) and maintained increased levels throughout the whole period of the experiment, reaching their maximum at 24 h FBE (16- and 9-fold higher than controls, respectively).

## 4. Discussion

In another experiment carried out by our research group [17] and focused on the ecophysiological and antioxidant responses of *S. officinalis* under a prolonged O_3_ exposure (120 ppb for 90 consecutive days, 5 h day^−1^), treated plants showed leaf yellowing and some disorders of leaf water status starting from 30 days FBE, suggesting that metabolic and cell ultrastructural impairments occurred. However, *S. officinalis* was able to alleviate the oxidative pressure and to defend itself by non-enzymatic antioxidant mechanisms, including carotenoids and phenols. In particular, we speculated that the reprogramming of metabolism to reroute carbon skeletons towards the synthesis of specialized metabolites represented an essential strategy in plant defence. However, the phenylpropanoid response of *S. officinalis* to chronic and acute O_3_ may differ [18]. Therefore, the first question we wanted to address was, “What is the potential role of phenolics (e.g., caffeic acid derivatives) and flavonoids in regulating the responses to a single O_3_ pulse?”

Variable O_3_-induced changes were observed over time among the detected phenylpropanoid compounds, likely because of their extraordinary functional diversity. Differences in phenolic profile, rather than in total phenolic content, could be crucial in sustaining plant defence. It is known that simple phenols such as caffeic acid protect in vivo and in vitro ascorbate peroxidase activity, which could provide cells with increased resistance against the formation of H_2_O_2_ in response to O_3_ treatment [25]. Here, the analysis of hydroxycinnamic acids revealed a significant increase of caffeic and ferulic acid contents starting from 2 h FBE, leading us to think about a possible high ability of these compounds to regulate the cellular redox state and to defend leaf tissues against O_3_, likely by modifying lignin composition (because of their antioxidant and barrier effects) [26]. By contrast, the levels of 3,4-dihydroxyphenyllactic acid, chlorogenic acid (only at 1 h FBE), rosmarinic acid, and salvianolic acid B significantly decreased during the exposure, suggesting that they might be directly decomposed by O_3_ or consumed by cells to counteract the accumulation of H_2_O_2_. The depletion of these phenolic compounds has been suggested to act as highly efficient radical scavenger, representing an important defence mechanism against the possible ROS generation due to increased oxidative metabolism [26,27]. At the recovery time (when all plants continued to appear visually symptomless), all examined caffeic acid derivatives (except chlorogenic acid) significantly increased, suggesting that stressed plants likely activated an additional antioxidant system capable of avoiding and scavenging ROS [6], although further analyses would be needed. The decrease of chlorogenic acid at 24 h FBE might be due to the involvement of this compound in coping with ascorbic acid destruction [28]. In addition, the analysis of hydroxybenzoic acids revealed a complex pattern of O_3_-driven changes, proposing the involvement of these specialized metabolites in promoting the allocation of plant resources to other metabolic processes. For example, benzoic acid could serve as precursor for salicylic acid and aromatic cytokinins, hormones involved in the cascade of signalling pathways triggered for enhancing plant defence [29].

Among the phenolic compounds, a major part of ROS scavenging activities is due to flavonoids as they show a wide localization in plant organs as well as different cells and cellular compartments. The number and arrangement of their hydroxyl groups attached to ring structures are important [30]. In the present study, we identified only two mono-B-ring-substituted flavonoids, i.e., the flavone apigenin and the flavanol kaempferol, both classified as “poor antioxidants” in comparison with other flavonoids [31]. These compounds showed different O_3_-induced responses; the apigenin content was significantly altered only during the first two hours of the O_3_ treatment, whereas kaempferol markedly changed starting from 2 h FBE. These secondary metabolites could be involved (i) in the capture of superoxide anions and reduction of cell membrane peroxidation, as confirmed by the decrease of apigenin and kaempferol observed at 1 and 2 h FBE, respectively; and (ii) in the regulation of H_2_O_2_ and prevention of hydroxyl radical production for stress signalling, as confirmed by the significant increase of apigenin observed at 2 h FBE and of kaempferol starting from the end of exposure [30].

Overall, the above-mentioned O_3_-induced variations of phenolic compounds were quite impressive given their magnitude, variability, and rapidity. However, considerable phenolic variations within few hours of exposure to different stresses were already reported in previous studies (e.g., [9,25,26,32,33]), although the phenolic responses (as well as other regulations) of plants to an O_3_ pulse remain understudied, at least in comparison with experiments focused on chronic O_3_ exposures [34,35]. Dramatic and variable phenolic changes reported in the present study, following metabolite-specific trends, were likely owing to the diversion of the investigated metabolites to other phenolic products as a result of specific changes in allocation within the different phenolic groups, as reported by Wellburn and Wellburn [36]. Specifically, flavonoids possess multiple physiological functions such as antioxidants, pathogen defense, insect attraction, rhizobium symbiosis, and auxin transport [37]. Moreover, a popular concept assumes that flavonoids are phytoalexins that can be inducibly synthesized by plants in response to pathogen and/or insect challenges [38]; and a single pulse of O_3_ is known to mimic a pathological agent by triggering a variety of defense reactions in plants that include the following: (i) rapid and transient increase of apoplastic ROS and (ii) induction of hypersensitive response [39]. Nevertheless, these results show that *S. officinalis* coping with a single O_3_ pulse was able to modify the phenylpropanoid pathway and likely take advantage of the specific defence functions associated to the array of their specialized metabolites. In particular, phenolics might be essential to regulate antioxidant defence and detoxification responses, either during or after the O_3_ treatment.

In light of the above, the second question was, “Which enzymes involved in the phenolic pathway are more affected by high O_3_ levels, and why?” It is known that the biosynthesis of phenolics under stressful conditions is regulated by the altered activities of various key enzymes of phenolic biosynthetic pathways like PAL (which catalyzes the initial step in the biosynthesis of plant phenolics and operates as a key regulatory enzyme in specialized metabolism) [40], PPO (which catalyzes the oxidation of monophenols and/or *o*-diphenols to *o*-quinones) [41], and RAS (which links PAL- and TAT-derived pathways to form RA as end product) [42]. In our study, an early decrease of all enzyme activities was observed during the first hours of treatment, probably due to an O_3_-induced oxidative damage to several proteins. This phenomenon occurs when the production of ROS exceeds the antioxidant defence mechanisms [43]. Both PAL and PPO activities were also suppressed at 24 h FBE, suggesting that these enzymes were probably not involved in defence response at the recovery time. Then, given the enhanced performance of RAS observed at the end of treatment and at the recovery time, it is possible to speculate that *S. officinalis* experiencing a single O_3_ pulse activated distinct pathways with different functions, likely to cope with potential future stress episodes [44]. This could be accompanied by the up- and downregulation of the transcript levels of genes encoding key biosynthetic enzymes in regulation of signal transduction systems and stress responses [4].

The third question was, “Which molecular alterations of gene expression related to the biosynthesis of RA are induced by O_3_ treatment?” Our results indicate that *PAL*, *RAS*, *C4H*, and *TAT* affect RA formation with varying degree and timing. This is in accordance with the study by Wang et al. [45], where 29 genes related to phenolic acid biosynthesis were identified in the genome of *S. miltiorrhiza*, ten of which are putatively involved in RA biosynthesis. In particular, the RA accumulation observed at 24 h FBE might be a result of the upregulation at different times of several co-expressed genes involved in RA biosynthesis (*PAL* and *RAS* at 5 h FBE; *C4H* and *TAT* throughout the entire experiment). In particular, the determination of transcript levels of genes encoding enzymes involved in RA biosynthesis by quantitative RT-PCR reflected the sequence of events taking place during and after the O_3_ treatment of *S. officinalis*. Following the decrease of both RA content, and PAL and RAS activities observed during O_3_ treatment, *PAL* and *RAS* genes were rapidly induced, as confirmed by upregulation of their expression at 5 h FBE. This regulation consequently resulted in the increase of RAS activity observed starting from the end of O_3_ treatment, as well as of RA content at 24 h FBE. As reported by Ejtahed et al. [46], no positive relation was observed between the expression intensity of *PAL* and the accumulation of RA, indicating that *PAL* is not the rate-determining step in RA biosynthesis and other unknown factors might participate in RA synthesis. It is worth noting that *PAL* genes have been cloned from different plant species where they usually exist in polygenic families, with three to nine members [47,48]. To date, we do not know how many *PAL* members are present in *S. officinalis*. In addition, an increased expression profile of genes encoding enzymes in the early part of the phenylpropanoid and flavonoid biosynthesis pathway (such as *C4H*) [49] and in the early section of RA biosynthesis (such as *TAT*) was observed throughout the whole experiment, suggesting that the mechanisms regulating proteins encoded by these genes were, at least in part, at transcriptional level, according to Gottardini et al. [50]. Song and Li [51] suggested that four genes (*PAL*, *C4H*, *4CL*, and *HPPR*) may serve as key genes in the regulation of rosmarinic acid accumulation in *S. miltiorrhiza*, while *TAT* may play an important role in regulating the content of salvianolic acid B. It is known that *TAT* is the first enzyme in the tyrosine-derived branch of RA biosynthesis. Its product, 4-hydroxyphenylpyruvic acid, serves as a precursor for homogentisic acid (catalyzed by hydroxyphenylpyruvate dioxygenase, HPPD), which is important for the formation of plastoquinones and tocopherols [52], as well as substrate for hydroxyphenylpyruvate reductase, the enzyme forming 4-hydroxyphenyllactic acid as precursor for RA [42]. Therefore, TAT activity is also required for the formation of other antioxidative compounds such as tocopherols [53].

## 5. Conclusions

In conclusion, *S. officinalis* faced the O_3_ pulse by regulating the activation and the timing of the phenolic biosynthetic route. Ozone-induced changes of phenylpropanoid profile were quite impressive in terms of magnitude, variability, and rapidity. This regulation was likely an integrated defence mechanism, although further research would be needed to reinforce the observed phenolic and flavonoid arrangements and confirm their involvement in coping O_3_ stress. Specifically, the performance of RAS was enhanced, accompanied by the upregulation of the transcript levels of genes encoding key biosynthetic enzymes, like *RAS* (only at 5 h FBE), *TAT*, and *C4H*. In this way, stressed plants were probably equipped with an additional ‘secondary’ antioxidant system capable of controlling the cascades of uncontrolled oxidation mechanisms and protecting plant cells from O_3_-induced oxidative damage. Further studies may be useful to investigate other genes coding for enzymes in branching points (and not directly involved in RA biosynthesis), where the flux might be redirected from the RA pathway (e.g., *CHS*) [54], in order to evaluate their role in RA accumulation.

## Figures and Tables

**Table 1:** Primers for housekeeping genes and specific primers for *Salvia officinalis*.

Primers	Sequences
*EF1α*	F: 5′-ACAACCCTGAGAAGATCCC-3′
	R: 5′-GCACAGTTCCAATACCACCAAT-3′
*Actin*	F: 5′-TCTCTTGACAGAAGCCCCTCT-3′
	R: 5′-GATGGGGACTGTATGGCTGA-3′
*PAL*	F: 5′-GAAGAACACCGTGAGCCAAG-3′
	R:5′-GCTCACACTCTTCTCCCCTT-3′
*C4H*	F: 5′-ATCTGAACCACCGCAACCTC-3′
	R: 5′-GTGAACACCATGTCCTGACC-3′
*TAT*	F: 5′-GCACCTAAAGAAGATTGCTGAG-3′
	R: 5′-ATTGATGACCAACCAACCAAGG-3′
*RAS*	F:5′-ACTACTTGAGGTCGTCGCTC-3′
	R:5′-CGGATTTGGCAGGAGATAGC-3′
